# Traumatic Spinal Cord Injury: An Overview of Pathophysiology, Models and Acute Injury Mechanisms

**DOI:** 10.3389/fneur.2019.00282

**Published:** 2019-03-22

**Authors:** Arsalan Alizadeh, Scott Matthew Dyck, Soheila Karimi-Abdolrezaee

**Affiliations:** Regenerative Medicine Program, Department of Physiology and Pathophysiology, Rady Faculty of Health Sciences, Spinal Cord Research Center, University of Manitoba, Winnipeg, MB, Canada

**Keywords:** spinal cord injury, secondary injury mechanisms, clinical classifications and demography, animal models, glial and immune response, glial scar, chondroitin sulfate proteoglycans (CSPGs), cell death

## Abstract

Traumatic spinal cord injury (SCI) is a life changing neurological condition with substantial socioeconomic implications for patients and their care-givers. Recent advances in medical management of SCI has significantly improved diagnosis, stabilization, survival rate and well-being of SCI patients. However, there has been small progress on treatment options for improving the neurological outcomes of SCI patients. This incremental success mainly reflects the complexity of SCI pathophysiology and the diverse biochemical and physiological changes that occur in the injured spinal cord. Therefore, in the past few decades, considerable efforts have been made by SCI researchers to elucidate the pathophysiology of SCI and unravel the underlying cellular and molecular mechanisms of tissue degeneration and repair in the injured spinal cord. To this end, a number of preclinical animal and injury models have been developed to more closely recapitulate the primary and secondary injury processes of SCI. In this review, we will provide a comprehensive overview of the recent advances in our understanding of the pathophysiology of SCI. We will also discuss the neurological outcomes of human SCI and the available experimental model systems that have been employed to identify SCI mechanisms and develop therapeutic strategies for this condition.

## Introduction

Spinal cord injury (SCI) is a debilitating neurological condition with tremendous socioeconomic impact on affected individuals and the health care system. According to the National Spinal Cord Injury Statistical Center, there are 12,500 new cases of SCI each year in North America (1). Etiologically, more than 90% of SCI cases are traumatic and caused by incidences such as traffic accidents, violence, sports or falls (2). There is a reported male-to-female ratio of 2:1 for SCI, which happens more frequently in adults compared to children (2). Demographically, men are mostly affected during their early and late adulthood (3rd and 8th decades of life) (2), while women are at higher risk during their adolescence (15–19 years) and 7th decade of their lives (2). The age distribution is bimodal, with a first peak involving young adults and a second peak involving adults over the age of 60 (3). Adults older than 60 years of age whom suffer SCI have considerably worse outcomes than younger patients, and their injuries usually result from falls and age-related bony changes (1).

The clinical outcomes of SCI depend on the severity and location of the lesion and may include partial or complete loss of sensory and/or motor function below the level of injury. Lower thoracic lesions can cause paraplegia while lesions at cervical level are associated with quadriplegia (4). SCI typically affects the cervical level of the spinal cord (50%) with the single most common level affected being C5 (1). Other injuries include the thoracic level (35%) and lumbar region (11%). With recent advancements in medical procedures and patient care, SCI patients often survive these traumatic injuries and live for decades after the initial injury (5). Reports on the clinical outcomes of patients who suffered SCI between 1955 and 2006 in Australia demonstrated that survival rates for those suffering from tetraplegia and paraplegia is 91.2 and 95.9%, respectively (5). The 40-year survival rate of these individuals was 47 and 62% for persons with tetraplegia and paraplegia, respectively (5). The life expectancy of SCI patients highly depends on the level of injury and preserved functions. For instance, patients with ASIA Impairment Scale (AIS) grade D who require a wheelchair for daily activities have an estimated 75% of a normal life expectancy, while patients who do not require wheelchair and catheterization can have a higher life expectancy up to 90% of a normal individual (6). Today, the estimated life-time cost of a SCI patient is $2.35 million per patient (1). Therefore, it is critical to unravel the cellular and molecular mechanisms of SCI and develop new effective treatments for this devastating condition. Over the past decades, a wealth of research has been conducted in preclinical and clinical SCI with the hope to find new therapeutic targets for traumatic SCI.

### An Overview of Primary Injury

SCI commonly results from a sudden, traumatic impact on the spine that fractures or dislocates vertebrae. The initial mechanical forces delivered to the spinal cord at the time of injury is known as primary injury where “displaced bone fragments, disc materials, and/or ligaments bruise or tear into the spinal cord tissue” (7–9). Notably, most injuries do not completely sever the spinal cord (10). Four main characteristic mechanisms of primary injury have been identified that include: (1) Impact plus persistent compression; (2) Impact alone with transient compression; (3) Distraction; (4) Laceration/transection (8, 11). The most common form of primary injury is impact plus persistent compression, which typically occurs through burst fractures with bone fragments compressing the spinal cord or through fracture-dislocation injuries (8, 12, 13). Impact alone with transient compression is observed less frequently but most commonly in hyperextension injuries (8). Distraction injuries occur when two adjacent vertebrae are pulled apart causing the spinal column to stretch and tear in the axial plane (8, 12). Lastly, laceration and transection injuries can occur through missile injuries, severe dislocations, or sharp bone fragment dislocations and can vary greatly from minor injuries to complete transection (8). There are also distinct differences between the outcomes of SCI in military and civilian cases. Compared to civilian SCI, blast injury is the common cause of SCI in battlefield that usually involves multiple segments of the spinal cord (14). Blast SCI also results in higher severity scores and is associated with longer hospital stays (15). A study on American military personnel, who sustained SCI in a combat zone from 2001 to 2009, showed increased severity and poorer neurological recovery compared to civilian SCI (15). Moreover, lower lumbar burst fractures and lumbosacral dissociation happen more frequently in combat injuries (1). Regardless of the form of primary injury, these forces directly damage ascending and descending pathways in the spinal cord and disrupt blood vessels and cell membranes (11, 16) causing spinal shock, systemic hypotension, vasospasm, ischemia, ionic imbalance, and neurotransmitter accumulation (17). To date, the most effective clinical treatment to limit tissue damage following primary injury is the early surgical decompression (< 24 h post-injury) of the injured spinal cord (18, 19). Overall, the extent of the primary injury determines the severity and outcome of SCI (20, 21).

### An Overview on Clinical Classification Systems for Spinal Cord Injury

Functional classification of SCI has been developed to establish reproducible scoring systems by which the severity of SCI could be measured, compared, and correlated with the clinical outcomes (20). Generally, SCI can be classified as either complete or incomplete. In complete SCI, neurological assessments show no spared motor or sensory function below the level of injury (4). In the past decades, several scoring systems have been employed for clinical classification of neurological deficits following SCI. The first classification system, “Frankel Grade,” was developed by Frankel and colleagues in 1969 (22). They assessed the severity and prognosis of SCI using numerical sensory and motor scales (22). This was a 5-grade system in which Grade A was the most severe SCI with complete loss of sensory and motor function below the level of injury. Grade B represented complete motor loss with preserved sensory function and sacral sparing. Patients in Grade C and D had different degrees of motor function preservation and Grade E represented normal sensory and motor function. The “Frankel Grade” was widely utilized after its publication due to its ease of use. However, lack of clear distinction between Grades C and D and inaccurate categorization of motor improvements in patients over time, led to its replacement by other scoring systems (20).

Other classification methods followed Frankel's system. In 1987, Bracken et al. at Yale University School of Medicine classified motor and sensory functions separately in a 5 and 7-scale systems, respectively (23). However, this scoring system failed to account for sacral function (20). Moreover, integration of motor and sensory classifications was impossible in this system and it was abandoned due to complexity and impracticality in clinical settings (20). Several other scoring systems were developed in 1970' and 1980's by different groups such as Lucas and Ducker at the Maryland Institute for Emergency Medical Services in late 1970's (24), Klose and colleagues at the University of Miami Neuro-spinal Index (UMNI) in early 1980s (25) and Chehrazi and colleagues (Yale Scale) in 1981 (26). These scoring systems also became obsolete due to their disadvantage in evaluation of sacral functions, difficulty of use or discrepancies between their motor and sensory scoring sub-systems (20).

### American Spinal Injury Association (ASIA) Scoring System

**Figure 1:**
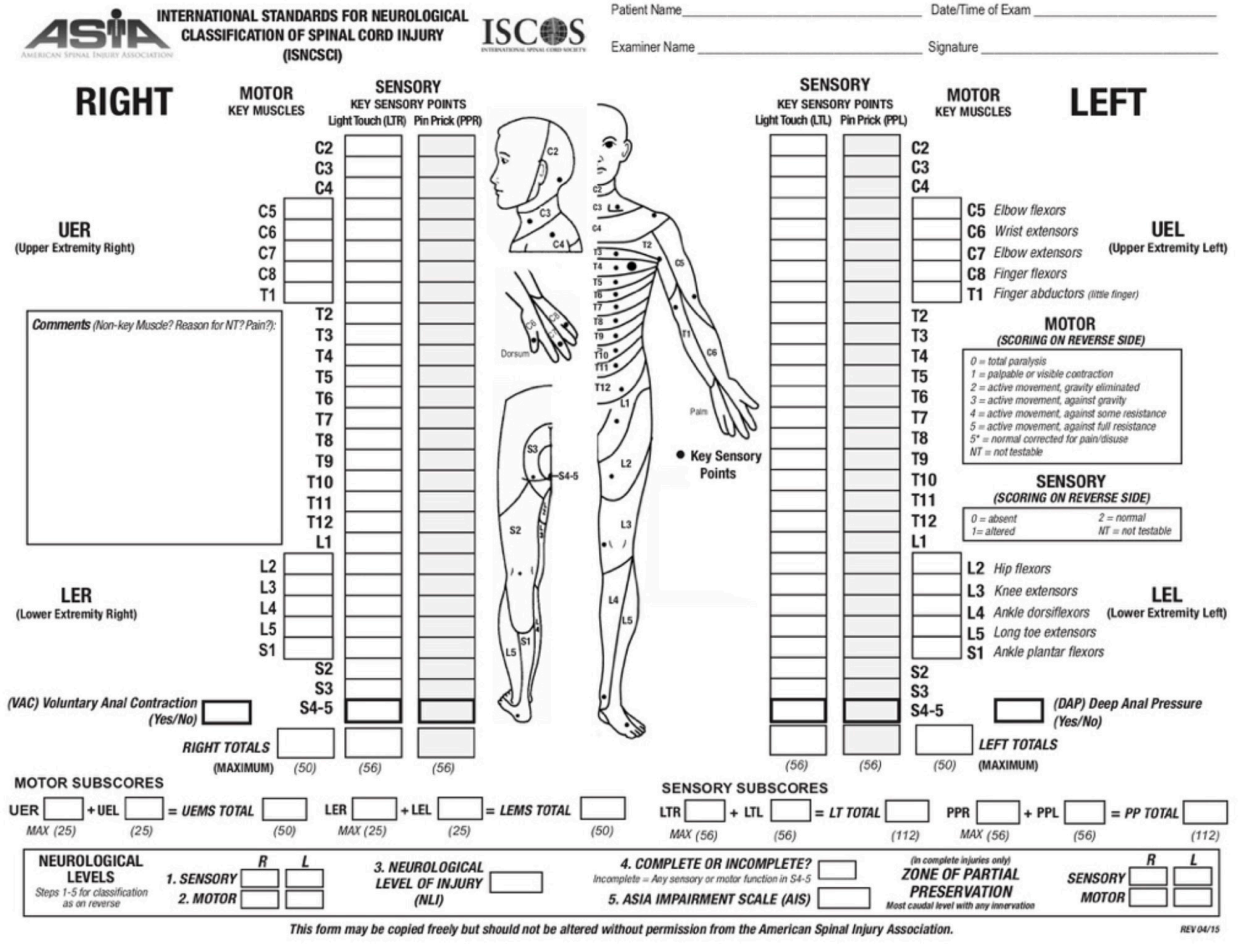
ASIA scoring for the neurological classification of the SCI. A sample scoring sheet used for ASIA scoring in clinical setting is provided (adopted from: http://asia-spinalinjury.org).

The ASIA scoring system is currently the most widely accepted and employed clinical scoring system for SCI. ASIA was developed in 1984 by the American Spinal Cord Injury Association and has been updated over time to improve its reliability (Figure 1). In this system, sensory function is scored from 0–2 and motor function from 0 to 5 (20). The ASIA impairment score (AIS) ranges from complete loss of sensation and movement (AIS = A) to normal neurological function (AIS = E). The first step in ASIA system is to identify the neurological level of injury (NLI). In this assessment, except upper cervical vertebrae that closely overlay the underlying spinal cord segments, the anatomical relationship between the spinal cord segments and their corresponding vertebra is not reciprocally aligned along the adult spinal cord (20). At thoracic and lumbar levels, each vertebra overlays a spinal cord segment one or two levels below and as the result, a T11 vertebral burst fracture results in neurological deficit at and below L1 spinal cord segment. Hence, the neurological level of injury (NLI) is defined as “the most caudal neurological level at which all sensory and motor functions are normal” (20). Upon identifying the NLI, if the injury is complete (AIS = A), “zone of partial preservation” (ZPP) is determined (20). ZPP is defined as all the segments below the NLI that have some preserved sensory or motor function. A precise record of ZPP enables the examiners to distinguish spontaneous from treatment-induced functional recovery, thus, essential for evaluating the therapeutic efficacy of treatments (20). Complete loss of motor and preservation of some sensory functions below the neurological level of the injury is categorized as AIS B (20). If motor function is also partially spared below the level of the injury, AIS score can be C or D (20). The AIS is scored D when the majority of the muscle groups below the level of the injury exhibit strength level of 3 or higher (for more details see Figure 1). ASIA classification combines the assessments of motor, sensory and sacral functions, thus addressing the shortcomings of previous scoring systems (20). The validity and reproducibility of ASIA system combined with its accuracy in prediction of patients' outcome have made it the most accepted and reliable clinical scoring system utilized for neurological classification of SCI (20).

### Neurological Outcomes of Spinal Cord Injury

In clinical management of SCI, neurological outcomes are generally determined at 72 h after injury using ASIA scoring system (20, 27). This time-point has shown to provide a more precise assessment of neurological impairments after SCI (28). One important predictor of functional recovery is to determine whether the injury was incomplete or complete. As time passes, SCI patients experience some spontaneous recovery of motor and sensory functions. Most of the functional recovery occurs during the first 3 months and in most cases reaches a plateau by 9 months after injury (20). However, additional recovery may occur up to 12–18 months post-injury (20). Long term outcomes of SCI are closely related to the level of the injury, the severity of the primary injury and progression of secondary injury, which will be discussed in this review.

Depending on the level of SCI, patients experience paraplegia or tetraplegia. Paraplegia is defined as the impairment of sensory or motor function in lower extremities (27, 28). Patients with incomplete paraplegia generally have a good prognosis in regaining locomotor ability (~76% of patients) within a year (27). Complete paraplegic patients, however, experience limited recovery of lower limb function if their NLI is above T9 (29). An NLI below T9 is associated with 38% chance of regaining some lower extremity function (29). In patients with complete paraplegia, the chance of recovery to an incomplete status is only 4% with only half of these patients regaining bladder and bowel control (29). Tetraplegia is defined as partial or total loss of sensory or motor function in all four limbs. Patients with incomplete tetraplegia will gain better recovery than complete tetra- and paraplegia (30). Unlike complete SCI, recovery from incomplete tetraplegia usually happens at multiple levels below the NLI (20). Patients generally reach a plateau of recovery within 9–12 months after injury (20). Regaining some motor function within the first month after the injury is associated with a better neurological outcome (20). Moreover, appearance of muscle flicker (a series of local involuntary muscle contractions) in the lower extremities is highly associated with recovery of function (31). Patients with complete tetraplegia, often (66–90%) regain function at one level below the injury (28, 30). Importantly, initial muscle strength is an important predictor of functional recovery in these patients (20). Complete tetraplegic patients with cervical SCI can regain antigravity muscle function in 27% of the cases when their initial muscle strength is 0 on a 5-point scale (32). However, the rate of regaining antigravity muscle strength at one caudal level below the injury increases to 97% when the patients have initial muscle strength of 1–2 on a 5-point scale (33).

An association between sensory and motor recovery has been demonstrated in SCI where spontaneous sensory recovery usually follows the pattern of motor recovery (20, 34). Maintenance of pinprick sensation at the zone of partial preservation or in sacral segments has been shown as a reliable predictor of motor recovery (35). One proposed reason for this association is that pinprick fibers in lateral spinothalamic tract travel in proximity of motor fibers in the lateral corticospinal tract, and thus, preservation of sensory fibers can be an indicator of the integrity of motor fiber (20). Diagnosis of an incomplete injury is of great importance and failure to detect sensory preservation at sacral segments results in an inaccurate assessment of prognosis (20).

## Experimental Models of Spinal Cord Injury

### An Overview of Available Animal Models

In the past few decades, various animal models have been developed to allow understanding the complex biomedical mechanisms of SCI and to develop therapeutic strategies for this condition. An ideal animal model should have several characteristics including its relevance to the pathophysiology of human SCI, reproducibility, availability, and its potential to generate various severities of injury (36).

Small rodents are the most frequently employed animals in SCI studies due to their availability, ease of use and cost-effectiveness compared to primates and larger non-primate models of SCI (36, 37). Among rodents, rats more closely mimic pathophysiological, electrophysiological, functional, and morphological features of non-primate and human SCI (38). In rat (39), cat (40), monkey (41), and human SCI (17), a cystic cavity forms in the center of the spinal cord, which is a surrounded by a rim of anatomically preserved white matter. A study by Metz and colleagues compared the functional and anatomical outcomes of rat contusive injuries and human chronic SCI (42). High resolution MRI assessments identified that SCI-induced neuroanatomical changes such as spinal cord atrophy and size of the lesion were significantly correlated with the electrophysiological and functional outcomes in both rat and human contusive injuries (42). Histological assessments in rats also showed a close correlation between the spared white matter and functional preservation following injury (42). These studies provide evidence that rat models of contusive SCI could serve as an adequate model to develop and evaluate the structural and functional benefits of therapeutic strategies for SCI (42).

Mice show different histopathology than human SCI in which the lesion site is filled with dense fibrous connective-like tissue (43–46). Mouse SCI studies show the presence of fibroblast-like cells expressing fibronectin, collagen, CD11b, CD34, CD13, and CD45 within the lesion core of chronic SCI, while it is absent in the injured spinal cord of rats (47). Another key difference between rat and mice SCI is the time-point of inflammatory cell infiltration. While microglia/macrophage infiltration is relatively consistent between rat and mouse models of SCI (47), there is a temporal difference in infiltration of neutrophils and T cells between the two species (47, 48). In SCI rats, infiltration of neutrophils, the first responders, peaks at 6 h post injury, followed by a significant decline at 24–48 h after SCI (48). Similarly, in mouse SCI, neutrophil infiltration occurs within 6 h following injury; however, their numbers continue to rise and do not peak until 3–14 days post injury (49). T cell infiltration also varies between rat and mouse SCI models (50). In rats, T cell infiltration occurs between 3 and 7 days post injury and declines by 50% in the following 2 weeks (47), whereas in mice, T cell infiltration is not detected until 14 days post injury and their number doubles between 2 and 6 weeks post injury (47). Regardless of their pathophysiological relevance, mice have been used extensively in SCI studies primarily due to the availability of transgenic and mutant mouse models that have allowed uncovering molecular and cellular mechanisms of SCI (38).

In recent years, there has been emerging interest in employment of non-human primates and other larger animals such as pig, dog and cat as intermediate pre-clinical models (51–53) to allow more effective translation of promising treatments from rodent models to human clinical trials (50). Although rodents have served as invaluable models for studying SCI mechanisms and therapeutic development, larger mammals, in particular non-human primates, share a closer size, neuroanatomy, and physiology to humans. Importantly, their larger size provides a more relevant platform for drug development, bioengineering inventions, and electrophysiological and rehabilitation studies. Nonetheless, both small and large animal models of SCI have limitations in their ability to predict the outcome in human SCI. One important factor is high degree of variability in the nature of SCI incidence, severity and location of the injury in human SCI, while in laboratory animal models, these variabilities are less (36). Values acquired by clinical scoring systems such as ASIA or Frankel scoring systems lack the consistency of the data acquired from laboratory settings, which makes the translation of therapeutic interventions from experimental to clinical settings challenging (36). A significant effect from an experimental treatment in consistent laboratory settings may not be reproducible in clinical settings due to high variability and heterogeneity in human populations and their injuries (36). To date, several pharmacological and cellular preclinical discoveries have led to human clinical trials based on their efficacy in improving the outcomes of SCI in small animal models. However, the majority of these trials failed to reproduce the same efficacy in human SCI. Thus, in pre-clinical studies, animal models, and study designs should be carefully chosen to reflect the reality of clinical setting as closely as possible (36). Larger animals provide the opportunity to refine promising therapeutic strategies prior to testing in human SCI; however, their higher cost, need for specialized facilities and small subject (sample) size have limited their use in SCI research (50). Thus, rodents are currently the most commonly employed models for preclinical discoveries and therapeutic development, while the use of larger animals is normally pursued for late stage therapies that have shown efficacy and promise in small animal models. Table 1 provides a summary of available SCI models.

**Table 1:** Summary of SCI models.

**References**	**Model**	**Species**	**Year developed**	**Mechanism**	**Pros**	**Cons**
Beattie et al. (54) Constantini et al. (55) Basso et al. (56)	MASCIS	Rodents	Early 1990s	Weight drop (10 g), contusion	Most widely used, impact velocity, compression distance, time, and rate are measurable	Bouncing effect causing double impact, inconsistent results
Scheff et al. (57)	IH Impactor	Rodents	Early 2000s	Controlled contusive impact	No bouncing, Graded injury severity	Learning curve
Stokes (58) Somerson and Stokes (59) Stokes and Somerson (60)	OSU/ESCID^*^	Rodents	Late 1980s/ Late 1990s	Controlled rapid contusive impact using an electromagnetic vibrator	Controlled displacement, reproducible, no bouncing, more similar to clinical SCI impact, precise, low variability	Complicated device setup, requires testing components, limited technical assistance
Rivlin and Tator (61) Joshi and Fehlings (62)	Clip compression	Rodents	Late 1970s	Modified aneurysm clip compression, compressive and contusive injury	Inexpensive, availability, simplicity, stabilization of spinal cord is not required, can inflict both contusion and compression injuries, different injury severity, clinically relevant	Need for calibration due to the loss of force after repeated use, difficult to reproduce consistent results between different operators, impact parameters not recordable
Marcol et al. (63)	Air-gun impactor	Rats	Early 2010s	Air pressure mediated contusion	Less invasive, no contact	Inconsistency, not validated, unable to produce graded injury severity
Blight (64) Plemel et al. (65)	Forceps compression	Guinea pig, rodents	Early 1990s	Compressive injury by a calibrated forceps	Bilateral compression, simple, inexpensive	Lack of accuracy, lack of contusion and compression, impact parameters not recordable not recordable
Tarlov and Klinger (66) Bao and Liu (67)	Balloon compression	Dogs, rats, primates, rabbits	Early 1950s	Compressive and contusive injury	Easy to perform	Inconsistency, impact parameters not recordable, lacks acute impact
da Costa et al. (68)	Spinal cord strapping	Rats	Late 2000s	Compressive injury using SC-strapper	Non-invasive, does not require laminectomy, graded injury possible, 100% survival rate	Inconsistency, not reproducible, not recordable
Choo et al. (12) Dabney et al. (69) Seifert et al. (70)	Harrington, UBC and UTA distractors	Rats	Early 2000s	Distraction	Resemblance to clinical scenarios	Inconsistency and complexity, not validated
Choo et al. (12) Fiford et al. (71)	Dislocation model	Rats	Early 2000s	Spinal dislocation	Resembles the clinical scenarios, no need for complex surgical procedures	Not validated, inconsistent
Kwon et al. (37) Heimburger (72)	Complete transection	A wide variety of small and large animals	1990s	Complete transection	Reproducible, consistent, easy to perform, useful for studying regeneration	Not clinically relevant
Dyer et al. (73) Seitz et al. (74) Inman et al. (75)	Partial transection	Same as above	1990s	Partial transection	Easier postoperative animal care compared to above, ideal for studying contra and ipsilateral lesions and plasticity	Inconsistency, not precise
Hall and Gregson (76) Dubois-Dalcq et al. (77) Matsushima and Morell (78) Woodruff and Franklin (79)	Chemical models	Rodents	Early 1970s onwards	Reagents such as ethidium bromide, lysolecithin, murine hepatitis virus, cuprizone, myelin specific antibodies and complement	Simple, allows for studying demyelination and remyelination	Inconsistency

* *ESCID, Electromagnetic SCI Device*.

### An Overview of Experimental Models of Spinal Cord Injury

Animal models are also classified based on the type of SCI. The following sections will provide an overview on the available SCI models that are developed based on injury mechanisms, their specifications and relevance to human SCI (Table 1).

#### Transection Models

A complete transection model of SCI is relatively easy to reproduce (51). However, this model is less relevant to human SCI as a complete transection of the spinal cord rarely happens (51). While they do not represent clinical reality of SCI, transection models are specifically suitable for studying axonal regeneration or developing biomaterial scaffolds to bridge the gap between proximal and distal stamps of the severed spinal cord (51). Due to complete disconnection from higher motor centers, this model is also suitable for studying the role of propriospinal motor and sensory circuits in recovery of locomotion following SCI (51, 80). Partial transection models including hemi-section, unilateral transection and dorsal column lesions are other variants of transection models (51). Partial transection models are valuable for investigation of nerve grafting, plasticity and where a comparison between injured and non-injured pathways is needed in the same animal (51). However, these models lead to a less severe injury and higher magnitude of spontaneous recovery rendering them less suitable for development and evaluation of new therapies (51).

#### Contusive Models

Contusion is caused by a transient physical impact to the spinal cord and is clinically-relevant. There are currently three types of devices that can produce contusion injury in animal models: weight-drop apparatus, electromagnetic impactor, and a recently introduced air gun device (51). The impactor model was first introduced by Gruner at New York University (NYU) in 1992 (81). The original NYU impactor included a metal rod of specific weight (10 g) that could be dropped on the exposed spinal cord from a specific height to induce SCI (51). This model allowed induction of a defined severity of SCI by adjusting the height, which the rod fell on the spinal cord (81). Parameters such as time, velocity at impact and biomechanical response of the tissue can be recorded for analysis and verification (51). The NYU impactor was later renamed to Multicenter Animal Spinal Cord Injury Study (MASCIS) impactor, and conditions surrounding the study and use of the MASCIS impactor were standardized (51). Since its introduction, the MASCIS impactor has been updated twice. The most recent version, MACIS III, was introduced in 2012 and included both electromagnetic control and digital recording of the impact parameters (51). However, inability to control duration of impact and “weight bounce,” that could cause multiple impacts, have been known limitations of MASCIS impactors (51).

The Infinite Horizon (IH) impactor is another type of impactor that utilizes a stepping motor to generate force-controlled impact in contrast to free fall in the MASICS impactor (51). This feature allows for better control over the force of impact and prevents “weight bounce” as the computer-controlled metal impounder can be immediately retracted upon transmitting a desired force to the spinal cord (51). IH impactor can be set to different force levels to provide mild, moderate and severe SCI in rats (ex. 100, 150, and 200 kdyn) (51). A limitation with IH impactors is unreliability of their clamps in holding the spinal column firmly during the impact that can cause inconsistent parenchymal injury and neurological deficits (51).

Ohio State University (OSU) impactor is a computer controlled electromagnetic impactor that was originally invented in 1987 and refined in 1992 to improve reliability (58). As the OSU impactor is electromagnetically controlled, multiple strikes are avoided (51). Subsequently, a modified version of the OSU impactor was developed in 2000 for use in mice (43). However, the OSU impactor is limited by its inability to determine the precise initial contact point with the spinal cord due to displacement of CSF upon loading the device (51). To date, MASCIS, IH and OSU impactor devices have been employed extensively and successfully to induce SCI. These impactor devices are available for small and large animals such as mice, rats, marmosets, cats, and pigs (51, 82).

#### Compressive Models

Compressive models of SCI have been also employed for several decades (61). While contusion injury is achieved by applying a force for a very brief period (milliseconds), the compression injury consists of an initial contusion for milliseconds followed by a prolonged compression through force application for a longer duration (seconds to minutes) (51). Thus, compression injury can be categorized as contusive-compressive models (51). Various models of compressive SCI are available.

Clip compression is the most commonly used compression model of SCI in rat and mice (51, 61, 62, 83). It was first introduced by Rivlin and Tator in 1978 (61). In this model, following laminectomy, a modified aneurism clip with a calibrated closing force is applied to the spinal cord for a specific duration of time (usually 1 min) to induce a contusive-compressive injury (51). The severity of injury can be calibrated and modified by adjusting the force of the clip and the duration of compression (51). For example, applying a 50 g clip for 1 min typically produces a severe SCI, while a 35 g clip creates a moderate to severe injury with the same duration (83). Aneurysm clips were originally designed for use in rat SCI, however, in recent years smaller and larger clips have been developed to accommodate its use in mice (62) and pig models (52). The clip compression model has several advantages compared to contusion models. This method is less expensive and easier to perform (51). Importantly, in contrast to the impactor injury that contusion is only applied dorsally to the spinal cord, the clip compression model provides contusion and compression simultaneously both dorsally and ventrally. Hence, clip compression model more closely mimics the most common form of human SCI, which is primarily caused by dislocation and burst compression fractures (83). Despite its advantages, clip compression model can create variabilities such as the velocity of closing and actual delivered force that cannot be measured precisely at the time of application (51).

Calibrated forceps compression has been also employed to induce SCI in rodents. This simple and inexpensive compressive model was first utilized in 1991 for induction of SCI in guinea pigs (64). In this method, a calibrated forceps with a spacer is used to compress the spinal cord bilaterally (51). This model lacks the initial impact and contusive injury, which is associated with most cases of human traumatic SCI. Accordingly, this model is not a clinically relevant model for reproducing human SCI pathology and therapeutic development (51).

Balloon Compression model has been also utilized extensively in primates and larger animals such as dogs and cats (84–86). In this model, a catheter with an inflatable balloon is inserted in the epidural or subdural space. The inflation of the balloon with air or saline for a specific duration of time provides the force for induction of SCI (51). Generally, all compression models (clip, forceps, and balloon) have the same limitation as the velocity and amount of force are unmeasurable (51).

In conclusion, while existing animal models do not recapitulate all clinical aspects of human SCI, the compression and contusion models are considered to be the most relevant and commonly employed methods for understanding the secondary injury mechanisms and therapeutic development for SCI.

### Overview of Secondary Mechanisms of Spinal Cord Injury

**Figure 2:**
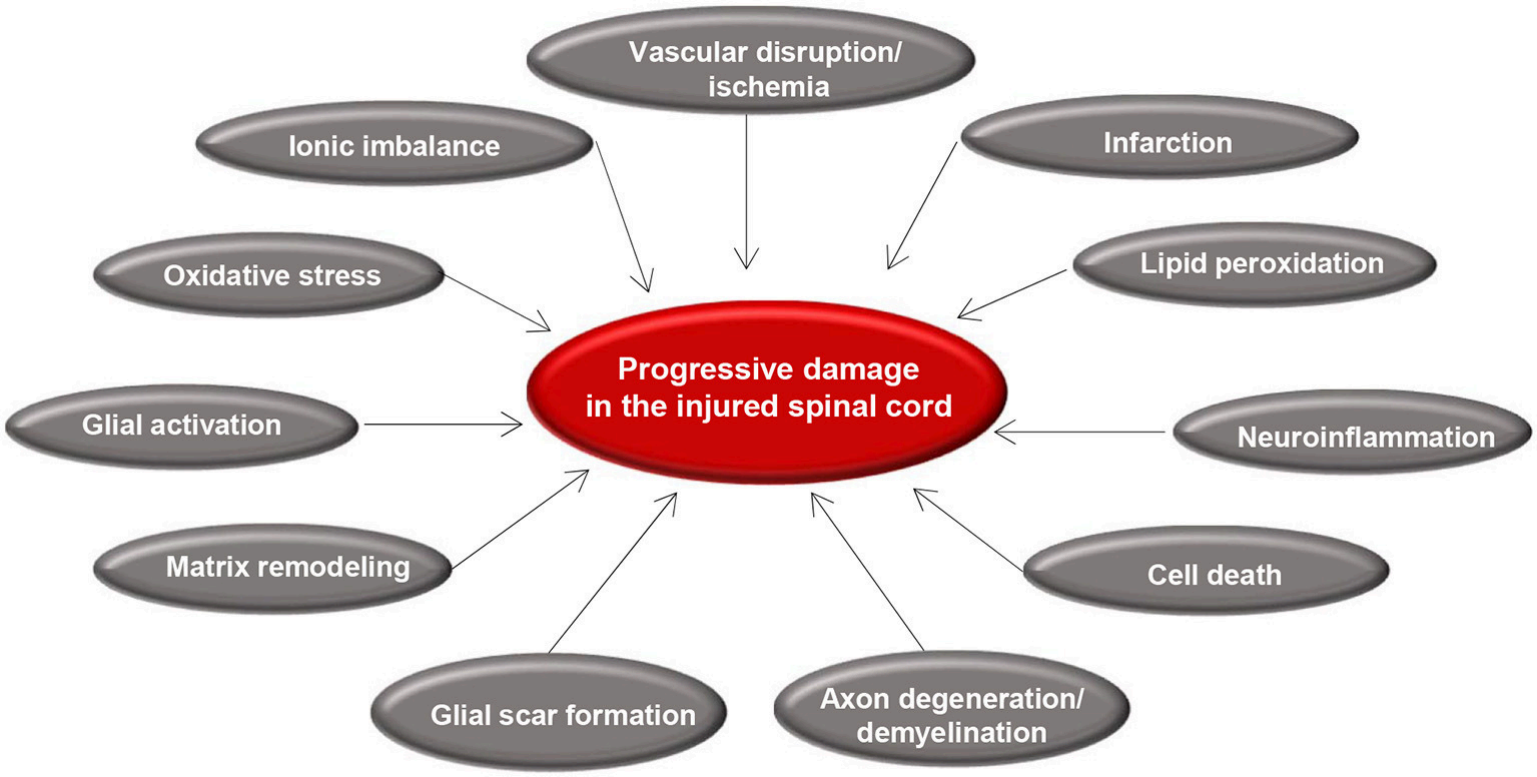
Summary of secondary injury processes following traumatic spinal cord injury. Diagram shows the key pathophysiological events that occur after primary injury and lead to progressive tissue degeneration. Vascular disruption and ischemia occur immediately after primary injury that initiate glial activation, neuroinflammation, and oxidative stress. These acute changes results in cell death, axonal injury, matrix remodeling, and formation of a glial scar.

Secondary injury begins within minutes following the initial primary injury and continues for weeks or months causing progressive damage of spinal cord tissue surrounding the lesion site (7). The concept of secondary SCI was first introduced by Allen in 1911 (87). While studying SCI in dogs, he observed that removal of the post traumatic hematomyelia improved neurological outcome. He hypothesized that presence of some “biochemical factors” in the necrotic hemorrhagic lesion causes further damage to the spinal cord (87). The term of secondary injury is still being used in the field and is referred to a series of cellular, molecular and biochemical phenomena that continue to self-destruct spinal cord tissue and impede neurological recovery following SCI (Figure 2) (20).

**Figure 3:**
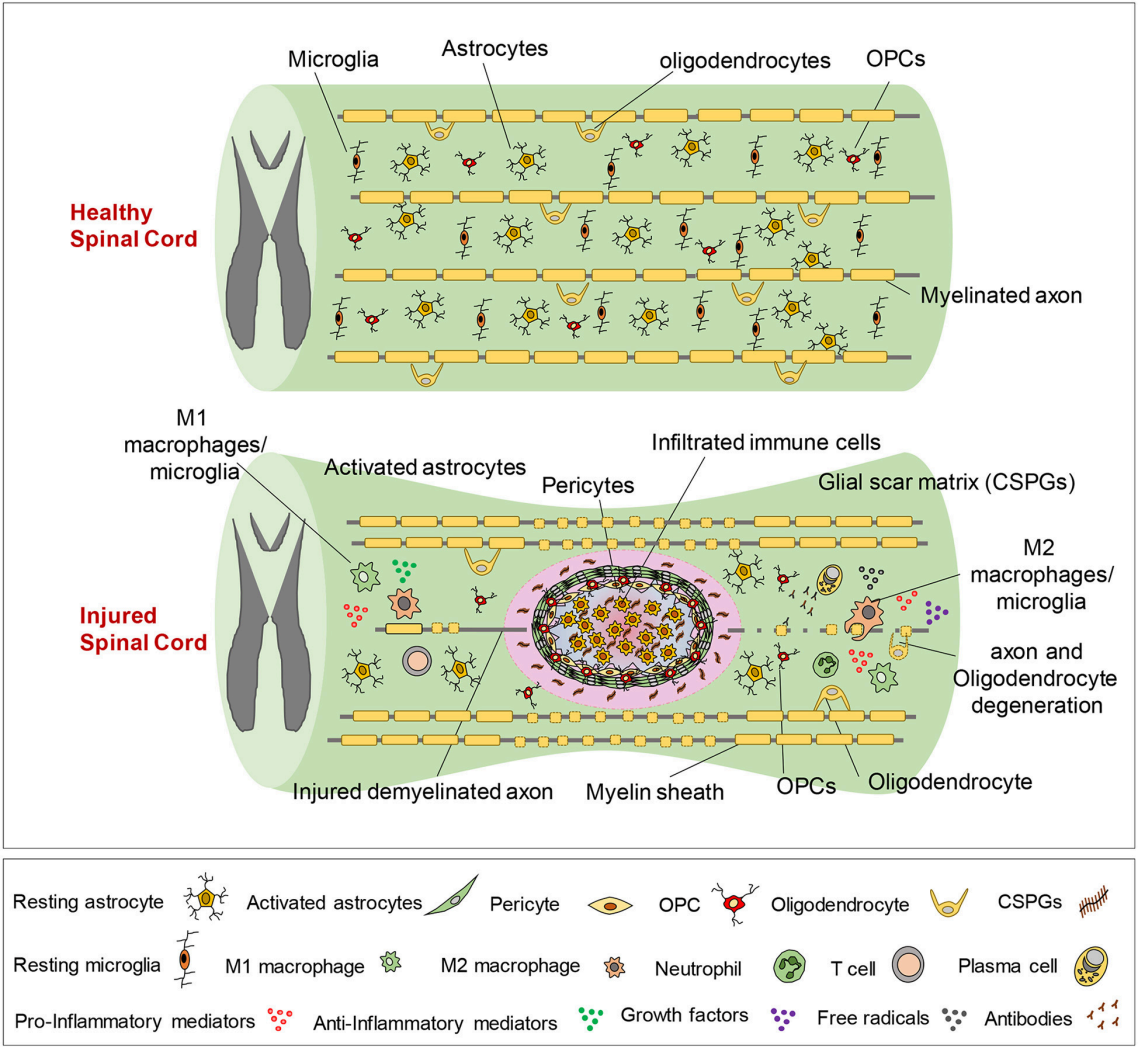
Pathophysiology of traumatic spinal cord injury. This schematic diagram illustrates the composition of normal and injured spinal cord. Of note, while these events are shown in one figure, some of the pathophysiological events may not temporally overlap and can occur at various phases of SCI, which are described here. Immediately after primary injury, activation of resident astrocytes and microglia and subsequent infiltration of blood-borne immune cells results in a robust neuroinflammatory response. This acute neuroinflammatory response plays a key role in orchestrating the secondary injury mechanisms in the sub-acute and chronic phases that lead to cell death and tissue degeneration, as well as formation of the glial scar, axonal degeneration and demyelination. During the acute phase, monocyte-derived macrophages occupy the epicenter of the injury to scavenge tissue debris. T and B lymphocytes also infiltrate the spinal cord during sub-acute phase and produce pro-inflammatory cytokines, chemokines, autoantibodies reactive oxygen and nitrogen species that contribute to tissue degeneration. On the other hand, M2-like macrophages and regulatory T and B cells produce growth factors and pro-regenerative cytokines such as IL-10 that foster tissue repair and wound healing. Loss of oligodendrocytes in acute and sub-acute stages of SCI leads to axonal demyelination followed by spontaneous remyelination in sub-acute and chronic phases. During the acute and sub-acute phases of SCI; astrocytes, OPCs and pericytes, which normally reside in the spinal cord parenchyma, proliferate and migrate to the site of injury and contribute to the formation of the glial scar. The glial scar and its associated matrix surround the injury epicenter and create a cellular and biochemical zone with both beneficial and detrimental roles in the repair process. Acutely, the astrocytic glial scar limits the spread of neuroinflammation from the lesion site to the healthy tissue. However, establishment of a mature longstanding glial scar and upregulation of matrix chondroitin sulfate proteoglycans (CSPGs) are shown to inhibit axonal regeneration/sprouting and cell differentiation in subacute and chronic phases.

Secondary injury can be temporally divided into acute, sub-acute, and chronic phases. The acute phase begins immediately following SCI and includes vascular damage, ionic imbalance, neurotransmitter accumulation (excitotoxicity), free radical formation, calcium influx, lipid peroxidation, inflammation, edema, and necrotic cell death (7, 20, 88). As the injury progresses, the sub-acute phase of injury begins which involves apoptosis, demyelination of surviving axons, Wallerian degeneration, axonal dieback, matrix remodeling, and evolution of a glial scar around the injury site (Figure 3). Further changes occur in the chronic phase of injury including the formation of a cystic cavity, progressive axonal die-back, and maturation of the glial scar (7, 89–92). Here, we will review the key components of acute secondary injury that contribute to the pathophysiology of SCI (Figures 2, 3).

### Vascular Injury, Ischemia and Hypoxia

Disruption of spinal cord vascular supply and hypo-perfusion is one of the early consequences of primary injury (93). Hypovolemia and hemodynamic shock in SCI patients due to excessive bleeding and neurogenic shock result in compromised spinal cord perfusion and ischemia (93). Larger vessels such as anterior spinal artery usually remain intact (94, 95), while rupture of smaller intramedullary vessels and capillaries that are susceptible to traumatic damage leads to extravasation of leukocytes and red blood cells (93). Increased tissue pressure in edematous injured spinal cord and hemorrhage-induced vasospasm in intact vessels further disrupts blood flow to the spinal cord (93, 95). In rat and monkey models of SCI, there is a progressive reduction in blood flow at the lesion epicenter within the first few hours after injury which remains low for up to 24 h (96). The gray matter is more prone to ischemic damage compared to the white matter as it has a 5-fold higher density of capillary beds and contains neurons with high metabolic demand (95, 97, 98). After injury, white matter blood flow typically returns to normal levels within 15 min post injury, whereas there are multiple hemorrhages in the gray matter and as a result, re-perfusion usually does not occur for the first 24 h (9, 99, 100). Vascular insult, hemorrhage and ischemia ultimately lead to cell death and tissue destruction through multiple mechanisms, including oxygen deprivation, loss of adenosine triphosphate (ATP), excitotoxicity, ionic imbalance, free radical formation, and necrotic cell death. Cellular necrosis and release of cytoplasmic content increase the extracellular level of glutamate causing glutamate excitotoxicity (93, 101). Moreover, re-establishment of blood flow in ischemic tissue leads to further damage through generating free radicals and eliciting an inflammatory response (93, 102) that will be discussed in this review.

### Ionic Imbalance, Excitotoxicity and Oxidative Damage

Within few minutes after primary SCI, the combination of direct cellular damage and ischemia/hypoxia triggers a significant rise of extracellular glutamate, the main excitatory neurotransmitter in the CNS (7). Glutamate binds to ionotropic (NMDA, AMPA, and Kainate receptors) as well as metabotropic receptors resulting in calcium influx inside the cells (103–105) (93). The effect of glutamate is not restricted to neurons as its receptors are vastly expressed on the surface of all glia and endothelial cells (103–106). Astrocytes can also release excess glutamate extracellularly upon elevation of their intracellular Ca^2+^ levels. Reduced ability of activated astrocytes for glutamate re-uptake from the interstitial space due to lipid peroxidation results in further accumulation of glutamate in the SCI milieu (93). Using microdialysis, elevated levels of glutamate have been detected in the white matter in the acute stage of injury (107). Based on a study by Panter and colleagues, glutamate increase is detected during the first 20–30 min post SCI and returns to the basal levels after 60 min (108).

Under normal condition, concentration of free Ca^2+^ can considerably vary in different parts of the cell (109). In the cytosol, Ca^2+^ ranges from 50–100 nM while it approaches 0.5–1.0 mM in the lumen of endoplasmic reticulum (110–112). A long-lasting abnormal increase in Ca^2+^ concentration in cytosol, mitochondria or endoplasmic reticulum has detrimental consequences for the cell (109–113). Mitochondria play a central role in calcium dependent neuronal death (113). In neurons, during glutamate induced excitotoxicity, NMDA receptor over-activity leads to mitochondrial calcium overload, which can cause apoptotic or necrotic cell death (113). Shortly after SCI, Ca^2+^ enters mitochondria through the mitochondrial calcium uniporter (MCU) (114). While the amount of mitochondrial calcium is limited during the resting state of a neuron, they can store a high amount of Ca^2+^ following stimulation (113). Calcium overload also activates a host of protein kinases and phospholipases that results in calpain mediated protein degradation and oxidative damage due to mitochondrial failure (93). In the injured white matter, astrocytes, oligodendrocytes and myelin are also damaged by the increased release of glutamate and Ca^2+^-dependent excitotoxicity (115). Within the first few hours after injury, oligodendrocytes show signs of caspase-3 activation and other apoptotic features, and their density declines (116). Interestingly, while glutamate excitotoxicity is triggered by ionic imbalance in the white matter, in the gray matter, it is largely associated with the activity of neuronal NMDA receptors (117, 118). Altogether, activation of NMDA receptors and consequent Ca^2+^ overload appears to induce intrinsic apoptotic pathways in neurons and oligodendrocytes and causes cell death in the first week of SCI in the rat (119, 120). Administration of NMDA receptor antagonist (MK-801) shortly following SCI has been associated with improved functional recovery and reduced edema (121).

Mitochondrial calcium overload also impedes mitochondrial respiration and results in ATP depletion disabling Na^+^/K^+^ ATPase and increasing intracellular Na^+^ (119, 122–124). This reverses the function of the Na^+^ dependent glutamate transporter that normally utilizes Na^+^ gradient to transfer glutamate into the cells (119, 125, 126). Moreover, the excess intracellular Na^+^ reverses the activity of Na^+^/Ca^2+^ exchanger allowing more Ca^+^ influx (127). Cellular depolarization activates voltage gated Na^+^ channels that results in entry of Cl^−^ and water into the cells along with Na^+^ causing swelling and edema (128). Increased Na^+^ concentration over-activates Na^+^/H^+^ exchanger causing a rise in intracellular H^+^ (101, 129). Resultant intracellular acidosis increases membrane permeability to Ca^2+^ that exacerbates the injury-induced ionic imbalance (101, 129). Axons are more susceptible to the damage caused by ionic imbalance due to their high concentration of voltage gated Na^+^ channels in the nodes of Ranvier (7). Accumulating evidence shows that administration of Na^+^ channel blockers such as Riluzole attenuates tissue damage and improves functional recovery in SCI underlining sodium as a key player in secondary injury mechanisms (130–133).

SCI results in production of free radicals and nitric oxide (NO) (114). Mitochondrial Ca^2+^ overload activates NADPH oxidase (NOX) and induces generation of superoxide by electron transport chain (ETC) (114). Reactive oxygen and nitrogen species (ROS and RNS) produced by the activity of NOX and ETC activates cytosolic poly (ADP ribose) polymerase (PARP). PARP consumes and depletes NAD^+^ causing failure of glycolysis, ATP depletion and cell death (114). Moreover, PAR polymers produced by PARP activity, induce the release of apoptosis inducing factor (AIF) from mitochondria and induce cell death (114). On the other hand, acidosis caused by SCI results in the release of intracellular iron from ferritin and transferrin (93). Spontaneous oxidation of Fe^2+^ to Fe^3+^ gives rise to more superoxide radicals (93). Subsequently, the Fenton reaction between Fe^3+^ and hydrogen peroxide produces highly reactive hydroxyl radicals (134). The resultant ROS and RNS react with numerous targets including lipids in the cell membrane with the most deleterious effects (93, 135). Because free radicals are short-lived and difficult to assess, measurements of their activity and final products, such as Malondialdehyde (MDA), are more reliable following SCI. Current evidence indicates that MDA levels are elevated as early as 1 h and up to 1 week after SCI (136, 137).

Oxidation of lipids and proteins is one of the key mechanisms of secondary injury following SCI (93). Lipid peroxidation starts when ROSs interact with polyunsaturated fatty acids in the cell membrane and generate reactive lipids that will then form lipid peroxyl radicals upon interacting with free superoxide radicals (138, 139). Each lipid peroxyl radical can react with a neighboring fatty acid, turn it into an active lipid and start a chain reaction that continues until no more unsaturated lipids are available or terminates when the reactive lipid quenches with another radical (93). The final products of this “termination” step of the lipid peroxidation is 4-hydroxynonenal (HNE) and 2-propenal, which are highly toxic to the cells (138–140). Lipid peroxidation is also an underlying cause of ionic imbalance through destabilizing cellular membranes such as cytoplasmic membrane and endoplasmic reticulum (93). Moreover, lipid peroxidation leads to Na^+^/K^+^ ATPase dysfunction that exacerbates the intracellular Na^+^ overload (141). In addition to ROS associated lipid peroxidation, amino acids are subject to significant RNS associated oxidative damage following SCI (93). RNSs (containing ONOO^−^) can nitrate the tyrosine residues of amino acids to form 3-nitrotyrosine (3-NT), a marker for peroxynitrite (ONOO^−^) mediated protein damage (139). Lipid and protein oxidation following SCI has a number of detrimental consequences at cellular level including mitochondrial respiratory and metabolic failure as well as DNA alteration that ultimately lead to cell death (141).

### Cell Death in Spinal Cord Injury

Cell death is a major event in the secondary injury mechanisms that affects neurons and glia after SCI (142–145). Cell death can happen through various mechanisms in response to various injury-induced mediators. Necrosis and apoptosis were originally identified as two major cell death mechanisms following SCI (146–148). However, recent research has uncovered additional forms of cell death. In 2012, the “Nomenclature Committee on Cell Death” (NCCD) NCCD defined 12 different forms of cell death such as necroptosis, pyroptosis, and netosis (149). Among the identified modes of cell death, to date, necrosis, necroptosis, apoptosis, and autophagy have been studied more extensively in the context of SCI and will be discussed in this review.

Following SCI, neurons and glial cells die through necrosis as the result of mechanical damage at the time of primary injury that also continues to the acute and subacute stages of injury (7, 150). Necrosis occurs due to a multitude of factors including accumulation of toxic blood components (151), glutamate excitotoxicity and ionic imbalance (152), ATP depletion (153), pro-inflammatory cytokine release by neutrophils and lymphocytes (154, 155), and free radical formation (142, 156–158). It was originally thought that necrosis is caused by a severe impact on a cell that results in rapid cell swelling and lysis. However, follow up evidence showed that in the case of seizure, ischemia and hypoglycemia, necrotic neurons show signs of shrunken, pyknotic, and condensed nuclei, with swollen, irreversibly damaged mitochondria and plasma membrane that are surrounded by astrocytic processes (159). Moreover, necrosis was conventionally viewed as instantaneous energy-independent non-programmed cell death (142, 156). However, recent research has identified another form of necrosis, termed as necroptosis, that is executed by regulated mechanisms.

Programmed necrosis or “necroptosis” has been described more recently as a highly regulated, caspase-independent cell death with similar morphological characteristics as necrosis (160). Necroptosis is a receptor-mediated process. It is induced downstream of the TNF receptor 1 (TNFR1) and is dependent on the activity of the receptor interacting protein kinase 1 (RIPK1) and RIPK3. Recent studies has uncovered a key role for RIPK1 as the mediator of necroptosis and a regulator of the innate immune response involved in both inflammation and cell death (161). Evidence from SCI studies show that lysosomal damage can potentiate necroptosis by promoting RIPK1 and RIPK3 accumulation (161). Interestingly, inhibition of necroptosis by necrostatin-1, a RIPK1 inhibitor, improves functional outcomes after SCI (150). These initial findings suggest that modulation of necroptosis pathways seems to be a promising target for neuroprotective strategies after SCI.

Apoptosis is the most studied mechanism of cell death after SCI. Apoptosis represents a programmed, energy dependent mode of cell death that begins within hours of primary injury (7). This process takes place in cells that survive the primary injury but endure enough insult to activate their apoptotic pathways (142). In apoptosis, the cell shrinks and is eventually phagocytosed without induction of an inflammatory response (156). Apoptosis typically occurs in a delayed manner in areas more distant to the injury site and most abundantly affects oligodendrocytes. In rat SCI, apoptosis happens as early as 4 h after the injury and reaches a peak at 7 day (156). At the site of injury majority of oligodendrocytes are lost within 7 days after SCI (162). However, apoptosis can be observed at a diminished rate for weeks after SCI (162, 163). Microglia and astrocytes also undergo apoptosis (156, 164). Interestingly, apoptotic cell death occurs in the chronically injured spinal cord in rat, monkey and human models of SCI, which is thought to be due to loss of trophic support from degenerating axons (146, 165).

Apoptosis is induced through extrinsic and intrinsic pathways based on the triggering mechanism (166). The extrinsic pathway is triggered by activation of death receptors such as FAS and TNFR1, which eventually activates caspase 8 (167). The intrinsic pathway, however, is regulated through a balance between intracellular pro- and anti-apoptotic proteins and is triggered by the release of cytochrome C from mitochondria and activating caspase 9 (167). In SCI lesion, apoptosis primarily happens due to injury induced Ca^2+^ influx, which activates caspases and calpain; enzymes involved in breakdown of cellular proteins (7). Moreover, it is believed that the death of neurons and oligodendrocytes in remote areas from the lesion epicenter can be mediated through cytokines such as TNF-α, free radical damage and excitotoxicity since calcium from damaged cells within the lesion barely reaches these remote areas (8, 168). Fas mediated cell death has been suggested as a key mechanism of apoptosis following SCI (144, 169–172). Post-mortem studies on acute and chronic human SCI and animal models revealed that Fas mediated apoptosis plays a role in oligodendrocyte apoptosis and inflammatory response at acute and subacute stages of SCI (173). Fas deficient mice exhibit a significant reduction in apoptosis and inflammatory response evidenced by reduced macrophage infiltration and inflammatory cytokine expression following SCI (173). Interestingly, Fas deficient mice show a significantly improved functional recovery after SCI (173) suggesting the promise of anti-apoptotic strategies for SCI.

SCI also results in a dysregulated autophagy (174). Normally, autophagy plays an important role in maintaining the homeostasis of cells by aiding in the turnover of proteins and organelles. In autophagy, cells degrade harmful, defective or unnecessary cytoplasmic proteins and organelles through a lysosomal dependent mechanism (175, 176). The process of autophagy starts with the formation of an autophagosome around the proteins and organelles that are tagged for autophagy (176). Next, fusion of the phagosome with a lysosome form an autolysosome that begins a recycling process (176). In response to cell injury and endoplasmic reticulum (ER) stress, autophagy is activated and limits cellular loss (177, 178). Current evidence suggests a neuroprotective role for autophagy after SCI (175, 179). Dysregulation of autophagy contributes to neuronal loss (174, 180). Accumulation of autophagosomes in ventral horn motor neurons have been detected acutely following SCI (181). Neurons with dysregulated autophagy exhibit higher expression of caspase 12 and become more prone to apoptosis (174). Moreover, blocking autophagy has been associated with neurodegenerative diseases such as Parkinson's and Alzheimer's disease (182–184). Autophagy promotes cell survival through elimination of toxic proteins and damaged mitochondria (185, 186). Interestingly, autophagy is crucial in cytoskeletal remodeling and stabilizes neuronal microtubules by degrading SCG10, a protein involved in microtubule disassembly (179). Pharmacological induction of autophagy in a hemi-section model of SCI in mice has been associated with improved neurite outgrowth and axon regeneration, following SCI (179). Altogether, although further studies are needed, autophagy is currently viewed as a beneficial mechanism in SCI.

### Adaptive and Innate Immune Response in Spinal Cord Injury

**Figure 4:**
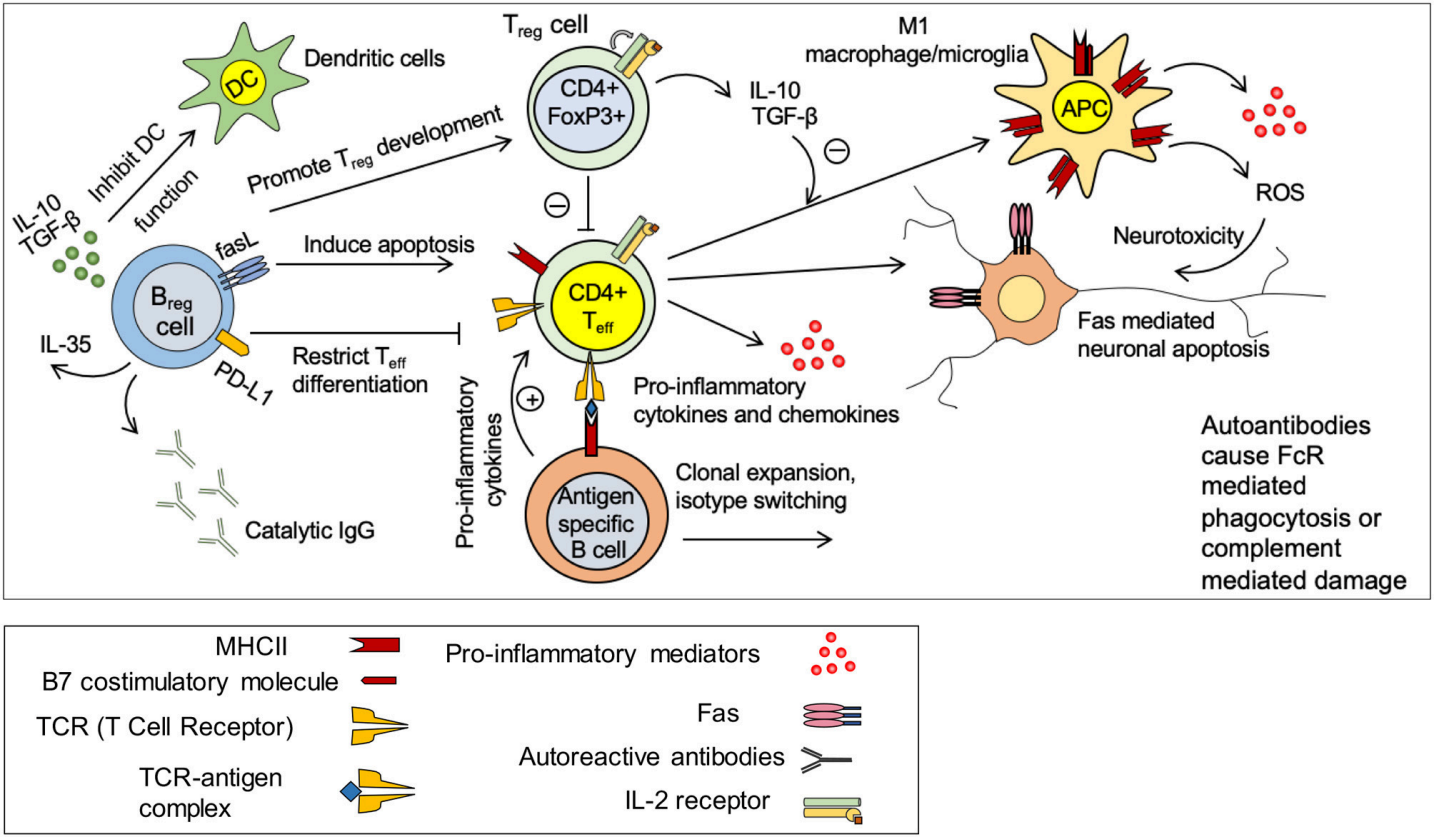
Immune response in spinal cord injury. Under normal circumstances, there is a balance between pro-inflammatory effects of CD4^+^ effector T cells (T_eff_) and anti-inflammatory effects of regulatory T and B cells (T_reg_ and B_reg_). T_reg_ and B_reg_ suppress the activation of antigen specific CD4^+^ T_eff_ cells through production of IL-10 and TGF-β. Injury disrupts this balance and promote a pro-inflammatory environment. Activated microglia/macrophages release pro-inflammatory cytokines and chemokines and present antigens to CD4^+^ T cells causing activation of antigen specific effector T cells. T_eff_ cells stimulate antigen specific B cells to undergo clonal expansion and produce autoantibodies against spinal cord tissue antigens. These autoantibodies cause neurodegeneration through FcR mediated phagocytosis or complement mediated cytotoxicity. M1 macrophages/microglia release pro-inflammatory cytokines and reactive oxygen species (ROS) that are detrimental to neurons and oligodendrocytes. B_reg_ cells possess the ability to promote T_reg_ development and restrict T_eff_ cell differentiation. B_reg_ cells could also induce apoptosis in T_eff_ cells through Fas mediate mechanisms.

Neuroinflammation is a key component of the secondary injury mechanisms with local and systemic consequences. Inflammation was originally thought to be detrimental for the outcome of SCI (187). However, now it is well-recognized that inflammation can be both beneficial and detrimental following SCI, depending on the time point and activation state of immune cells (188). There are multiple cell types involved in the inflammatory response following injury including neutrophils, resident microglia, and astrocytes, dendritic cells (DCs), blood-born macrophages, B- and T-lymphocytes (189) (Figure 4). The first phase of inflammation (0–2 days post injury) involves the recruitment of resident microglia and astrocytes and blood-born neutrophils to the injury site (190). The second phase of inflammation begins approximately 3 days post injury and involves the recruitment of blood-born macrophages, B- and T-lymphocytes to the injury site (189, 191–193). T lymphocytes become activated in response to antigen presentation by macrophages, microglia and other antigen presenting cells (APCs) (194). CD4^+^ helper T cells produce cytokines that stimulate B cell antibody production and activate phagocytes (195) (Figure 4). In SCI, B cells produce autoantibodies against injured spinal cord tissue, which exacerbate neuroinflammation and cause tissue destruction (196). While inflammation is more pronounced in the acute phase of injury, it continues in subacute and chronic phase and may persist for the remainder of a patients' life (193). Interestingly, composition and phenotype of inflammatory cells change based on the injury phase and the signals present in the injury microenvironment. It is established that microglia/macrophages, T cells, B cells are capable of adopting a pro-inflammatory or an anti-inflammatory pro-regenerative phenotype in the injured spinal cord (191, 197–199). The role of each immune cell population in the pathophysiology of SCI will be discussed in detail in upcoming sections.

#### Astrocytes

Astrocytes are not considered an immune cell *per se*; however, they play pivotal roles in the neuroinflammatory processes in CNS injury and disease. Their histo-anatomical localization in the CNS has placed them in a strategic position for participating in physiological and pathophysiological processes in the CNS (200). In normal CNS, astrocytes play major roles in maintaining CNS homeostasis. They contribute to the structure and function of blood-brain-barrier (BBB), provide nutrients and growth factors to neurons (200), and remove excess fluid, ions, and neurotransmitters such as glutamate from synaptic spaces and extracellular microenvironment (200). Astrocytes also play key roles in the pathologic CNS by regulating BBB permeability and reconstruction as well as immune cell activity and trafficking (201). Astrocytes contribute to both innate and adaptive immune responses following SCI by differential activation of their intracellular signaling pathways in response to environmental signals (201).

Astrocytes react acutely to CNS injury by increasing cytokine and chemokine production (202). They mediate chemokine production and recruitment of neutrophils through an IL-1R1-Myd88 pathway (202). Activation of the nuclear factor kappa b (NF-κB) pathway, one of the key downstream targets of interleukin (IL)1R-Myd88 axis, increases expression of intracellular adhesion molecule (ICAM) and vascular cell adhesion molecule (VCAM), which are necessary for adhesion and extravasation of leukocytes in inflammatory conditions such as SCI (201, 202). Within minutes of injury, production of IL-1β is significantly elevated in astrocytes and microglia (203). Moreover, chemokines such as monocyte chemoattractant protein (MCP)-1, chemokine C-C motif ligand 2 (CCL2), C-X-C motif ligand 1 (CXCL1), and CXCL2 are produced by astrocytes, and enhance the recruitment of neutrophils and pro-inflammatory macrophages following injury (201, 202). Astrocytes also promote pro-inflammatory M1-like phenotype in microglia/macrophages in the injured spinal cord through their production of TNF-α, IL-12, and IFN-γ (204–206). Interestingly, astrocytes also produce anti-inflammatory cytokines, such as TGF-β and IL-10, which can promote a pro-regenerative M2-like phenotype in microglia/macrophages (201, 207, 208).

Immunomodulatory role of astrocytes is defined by activity of various signaling pathways through a wide variety of surface receptors (200). For example, gp130, a member of IL-6 cytokine family, activates SHP2/Ras/Erk signaling cascade in astrocytes and limits neuroinflammation in autoimmune rodent models (209). TGF-β signaling in astrocytes has been implicated in modulation of neuroinflammation through inhibition of NF-κB activity and nuclear translocation (201, 210). STAT3 is another key signaling pathway in astrocytes with beneficial properties in neuroinflammation. Increase in STAT3 phosphorylation enhances astrocytic scar formation and restricts the expansion of inflammatory cells in mouse SCI, which is associated with improved functional recovery (211). Detrimental signaling pathways in astrocytes are known to be activated by cytokines, sphingolipids and neurotrophins (200). As an example, IL-17 is a key pro-inflammatory cytokine produced by effector T cells that can bind to IL-17R on the astrocyte surface (200). Activation of IL-17R results in the activation of NF-κB, which enhances expression of pro-inflammatory mediators, activation of oxidative pathways and exacerbation of neuroinflammation (200, 212). This evidence shows the significance of astrocytes in the inflammatory processes following SCI and other neuroinflammatory diseases of the CNS.

#### Neutrophils

Neutrophils infiltrate the spinal cord from the bloodstream within the first few hours after injury (213). Their population increases acutely in the injured spinal cord tissue and reaches a peak within 24 h post-injury (214). The presence of neutrophils is mostly limited to the acute phase of SCI as they are rarely found sub-acutely in the injured spinal cord (214). The role of neutrophils in SCI pathophysiology is controversial. Evidence shows that neutrophils contribute to phagocytosis and clearance of tissue debris (48). They release inflammatory cytokines, proteases and free radicals that degrade ECM, activate astrocytes and microglia and initiate neuroinflammation (48). Although neutrophils have been conventionally associated with tissue damage (48, 215), their elimination compromises the healing process and impedes functional recovery (216).

To elucidate the role of neutrophils in SCI, Stirling and colleagues used a specific antibody to reduce circulating LyG6/Gr1^+^ neutrophils in a mouse model of thoracic contusive SCI (216). This approach significantly reduced neutrophil infiltration in the injured spinal cord by 90% at 24 and 48 h after SCI (216). Surprisingly, neutrophil depletion aggravated the neurological and structural outcomes in the injured animals suggesting a beneficial role for neutrophils in the acute phase of injury (216). It is shown that simulated neutrophils release IL-1 receptor antagonist that can exert neuroprotective effects following SCI (217). Moreover, ablation of neutrophils results in altered expression of cytokines and chemokines and downregulation of growth factors such as fibroblast growth factors (FGFs), vascular endothelial growth factors (VEGFs) and bone morphogenetic proteins (BMPs) in the injured spinal cord that seemingly disrupt the normal healing process (216). Altogether, neutrophils play important roles in regulating neuroinflammation at the early stage of SCI that shapes the immune response and repair processes at later stages. While neutrophils were originally viewed as being detrimental in SCI, emerging evidence shows their critical role in the repair process. Further investigations are required to elucidate the role of neutrophils in SCI pathophysiology.

#### Microglia and Macrophages

Following neutrophil invasion, microglia/macrophages populate the injured spinal cord within 2–3 days post-SCI. Macrophage population is derived from invading blood-borne monocytes or originate from the CNS resident macrophages that reside in the perivascular regions within meninges and subarachnoid space (218, 219). The population of microglia/macrophages reaches its peak at 7–10 days post-injury in mouse SCI, followed by a decline in the subacute and chronic phases (20, 220). While macrophages and microglia share many functions and immunological markers, they have different origins. Microglia are resident immune cells of the CNS that originate from yolk sac during the embryonic period (221). Macrophages are derived from blood monocytes, which originate from myeloid progeny in the bone marrow (222, 223). Upon injury, acute disruption of brain-spinal cord barrier (BSB) enables monocytes, to infiltrate the spinal cord tissue and transform into macrophages (222). Macrophages populate the injury epicenter, while resident microglia are mainly located in the perilesional area (222). Once activated, macrophages, and microglia are morphologically and immunohistologically indistinguishable (224). Macrophages and microglia play a beneficial role in CNS regeneration. They promote the repair process by expression of growth promoting factors such as nerve growth factor (NGF), neurotrophin-3 (NT-3) and thrombospondin (225, 226). Macrophages and microglia are important for wound healing process following SCI due to their ability for phagocytosis and scavenging damaged cells and myelin debris following SCI (222, 227).

Based on microenvironmental signals, macrophages/microglia can be polarized to either pro-inflammatory (M1-like) or anti-inflammatory pro-regenerative (M2-like) phenotype, and accordingly contribute to injury or repair processes following SCI (191, 224, 228–230). Whether both microglia and macrophages possess the ability to polarize or it is mainly the property of monocyte derived macrophages is still a matter of debate and needs further elucidation (231–233). Some evidence show that Proinflammatory M1-like microglia/macrophages can be induced by exposure to T_h_1 specific cytokine, interferon (IFN)-γ (224, 230). Moreover, the SCI microenvironment appears to drive M1 polarization of activated macrophages (231). SCI studies have revealed that increased level of the proinflammatory cytokine, TNF-α, and intracellular accumulation of iron drives an M1-like proinflammatory phenotype in macrophages after injury (231). Importantly, following SCI, activated M1-like microglia/macrophages highly express MHCII and present antigens to T cells and contribute to the activation and regulation of innate and adaptive immune response (Figure 4) (224, 228). Studies on acute and subacute SCI and experimental autoimmune encephalomyelitis (EAE) models have shown that M1-like macrophages are associated with higher expression of chondroitin sulfate proteoglycans (CSPGs) and increased EAE severity and tissue damage (234–237). *In vitro*, addition of activated M1-like macrophages to dorsal root ganglion (DRG) neuron cultures leads to axonal retraction and failure of regeneration as the expression of CSPGs is much higher in M1-like compared to M2-like macrophages (237, 238). M1-like macrophages also produce other repulsive factors such as repulsive guidance molecule A (RGMA) that is shown to induce axonal retraction following SCI (239, 240). Interestingly, recent evidence shows that IFN-γ and TNFα polarized M1 microglia show reduced capacity for phagocytosis (241), a process that is critical for tissue repair after SCI.

Pro-regenerative M2-like microglia/macrophages, are polarized by T_h_2 cytokines, IL-4 and IL-13 and exhibit a high level of IL-10, TGF-β, and arginase-1 with reduced NF-κB pathway activity (224). IL-10 is a potent immunoregulatory cytokine with positive roles in repair and regeneration following CNS injury (242–244). IL-10 knock-out mice show higher production of pro-inflammatory and oxidative stress mediators after SCI (245). Lack of IL-10 is also correlated with upregulated levels of pro-apoptotic factors such as Bax and reduced expression of anti-apoptotic factors such as Bcl-2 (245). SCI mice that lacked IL-10 exhibited poorer recovery of function compared to wild-type mice (245). Our recent studies show that IL-10 polarized M2 microglia show enhanced capacity for phagocytosis (241). We have also found that M2 polarized microglia enhance the ability of neural precursor cells for oligodendrocyte differentiation through IL-10 mediated mechanisms (241). In addition to immune modulation, M2-like microglia/macrophages promote axonal regeneration (224). However, similar to the detrimental effects of prolonged M1 macrophage response, excessive M2-like activity promotes fibrotic scar formation through the release of factors such as TGF-β, PDGF, VEGF, IGF-1, and Galectin-3 (224, 246–248). Hence, a balance between proinflammatory M1 and pro-regenerative M2 macrophage/microglia response is beneficial for the repair of SCI (249).

#### T and B Lymphocytes

T and B lymphocytes play pivotal role in the adaptive immune response after SCI (194). Lymphocytes infiltrate the injured spinal cord acutely during the first week of injury and remain chronically in mouse and rat SCI (47, 193, 194, 196). In contrast to the innate immune response that can be activated directly by foreign antigens, the adaptive immune response requires a complex signaling process in T cells elicited by antigen presenting cells (250). Similar to other immune cells, T and B lymphocytes adopt different phenotypes and contribute to both injury and repair processes in response to microenvironmental signals (194, 251). SCI elicits a CNS-specific autoimmune response in T and B cells, which remains active chronically (196). Autoreactive T cells can exert direct toxic effects on neurons and glial cells (194, 252). Moreover, T cells can indirectly affect neural cell function and survival through pro-inflammatory cytokine and chemokine production (e.g. IL-1β, TNF-α, IL-12, CCL2, CCL5, and CXCL10) (194, 252). Genetic elimination of T cells (in athymic nude rats) or pharmacological inhibition of T cells (using cyclosporine A and tacrolimus) leads to improved tissue preservation and functional recovery after SCI (194, 253) signifying the impact of T cells in SCI pathophysiology and repair.

Under normal circumstances, systemic autoreactive effector CD4^+^ helper T cells (T_eff_) are suppressed by CD4^+^FoxP3^+^ regulatory T cells (T_reg_) (Figure 4) (194, 254). This inhibition is regulated through various mechanisms such as release of anti-inflammatory cytokines IL-10 and TGF-β by the T_reg_ cells (Figure 4) (194). Moreover, it is known that T_reg_ mediated inhibition of antigen presentation by dendritic cells (DCs) prevent T_eff_ cell activation (194). Following SCI, this T_reg_ -T_eff_ regulation is disrupted. Increased activity of autoreactive T_eff_ cells contributes to tissue damage through production of pro-inflammatory cytokines and chemokines, promoting M1-like macrophage phenotype and induction of Fas mediated neuronal and oligodendroglial apoptosis (Figure 4) (173). Moreover, autoreactive T_eff_ cells promote activation and differentiation of antigen specific B cells to autoantibody producing plasma cells that contribute to tissue damage after SCI (255). In SCI and MS patients, myelin specific proteins such as myelin basic protein (MBP) significantly increase the population of circulating T cells (256, 257). Moreover, serological assessment of SCI patients has shown high levels of CNS reactive IgM and IgG isotypes confirming SCI-induced autoimmune activity of T and B cells (Figure 4) (196, 258, 259). In animal models of SCI, serum IgM level increases acutely followed by an elevation in the levels of IgG1 and IgG2a at later time-points (196). In addition to autoantibody production, autoreactive B cells contribute to CNS injury through pro-inflammatory cytokines that stimulate and maintain the activation states of T_eff_ cells (194, 260). B cell knockout mice (BCKO) that have no mature B cell but with normal T cells, show a reduction in lesion volume, lower antibody levels in the cerebrospinal fluid and improved recovery of function following SCI compared to wild-type counterparts (255). Of note, antibody mediated injury is regulated through complement activation as well as macrophages/microglia that express immunoglobulin receptors (193, 255).

The effect of SCI on systemic B cell response is controversial. Evidence shows that SCI can suppress B cell activation and antibody production (261). Studies in murine SCI have shown that B cell function seems to be influenced by the level of injury (262). While injury to upper thoracic spinal cord (T3) suppresses the antibody production, a mid-thoracic (T9) injury has no effect on B cell antibody production (262). An increase in the level of corticosterone in serum together with elevation of splenic norepinephrine found to be responsible for the suppression of B cell function acutely following SCI (261). Elevated corticosterone and norepinephrine leads to upregulation of lymphocyte beta-2 adrenergic receptors eliciting lymphocyte apoptosis (194). This suggests a critical role for sympathetic innervation of peripheral lymphoid tissues in regulating B cell response following CNS injury (261). Despite their negative roles, B cells also contribute to spinal cord repair following injury through their immunomodulatory B_reg_ phenotype (Figure 4) (263). B_reg_ cells control antigen-specific T cell autoimmune response through IL-10 production (264).

Detrimental effects of SCI-induced autoimmunity are not limited to the spinal cord. Autoreactive immune cells contribute to the exacerbation of post-SCI sequelae such as cardiovascular, renal and reproductive dysfunctions (194). For example, presence of an autoantibody against platelet prostacyclin receptor has been associated with a higher incidence of coronary artery disease in SCI patients (265). Collectively, evidence shows the critical role of adaptive immune system in SCI pathophysiology and repair. Thus, treatments that harness the pro-regenerative properties of the adaptive immune system can be utilized to reduce immune mediated tissue damage, improve neural tissue preservation and facilitate repair following SCI.

### Glial Scar and Extracellular Matrix

Traumatic SCI triggers the formation of a glial scar tissue around the injury epicenter (266, 267). The glial scar is a multifactorial phenomenon that is contributed f several populations in the injured spinal cord including activated astrocytes, NG2^+^ oligodendrocyte precursor cells (OPCs), microglia, fibroblasts, and pericytes (268–271). The heterogeneous scar forming cells and associated ECM provides a cellular and biochemical zone within and around the lesion (Figure 3) (272). Resident and infiltrating inflammatory cells contribute to the process of glial activation and scar formation by producing cytokines (e.g., IL-1β and IL-6) chemokines and enzymes that activate glial cells or disrupt BSB (267). Activated microglia/macrophages produce proteolytic enzymes such as matrix metalloproteinases (MMPs) that increase vascular permeability and further disruption of the BSB (273). Inhibition of MMPs improves neural preservation and functional recovery in animal models of SCI (273–275). In addition to glial and immune cells, fibroblasts, pericytes and ependymal cells also contribute to the structure of the glial scar (267). In penetrating injuries where meninges are compromised, meningeal fibroblasts infiltrate the lesion epicenter (276). Fibroblasts contribute to the production of fibronectin, collagen, and laminin in the ECM of the inured spinal cord (267) and are a source of axon-repulsing molecules such as semaphorins that influence axonal regeneration following SCI (277). Fibroblasts have also been found in contusive injuries where meninges are intact (268, 270). Studies using genetic fate mapping in these injuries have unraveled that perivascular pericytes and fibroblasts migrate to the injury site and form a fibrotic core in the scar which matures within 2 weeks post-injury (268, 270). SCI also triggers proliferation and migration of the stem/progenitor cell pool of the spinal cord parenchyma and ependyma. These cells can give rise to new scar forming astrocytes and OPCs (278–280). In a mature glial scar, activated microglia/macrophages occupy the innermost portion closer to the injury epicenter surrounded by NG2^+^ OPCs (Figure 3) (267), while reactive astrocytes reside in the injury penumbra and form a cellular barrier (267). Of note, in human SCI, the glial scar begins to form within the first hours after the SCI and remains chronically in the spinal cord tissue (281). The glial scar has been found within the injured human spinal cord up to 42 years after the injury (267).

Activated astrocytes play a leading role in the formation of the glial scar (267). Following injury, astrocytes increase their expression of intermediate filaments, GFAP, nestin and vimentin, and become hypertrophied (282, 283). Reactive astrocytes proliferate and mobilize to the site of injury and form a mesh like structure of intermingled filamentous processes around the injury epicenter (284, 285). The astrocytic glial scar has been shown to serve as a protective barrier that prevents the spread of infiltrating immune cells into the adjacent segments (267, 284, 286). Attenuating astrocyte reactivity and scar formation by blockade of STAT3 activation results in poorer outcomes in SCI (211, 286). Reactive astrogliosis is also essential for reconstruction of the BBB, and blocking this process leads to exacerbated leukocyte infiltration, cell death, myelin damage, and reduced functional recovery (211, 285, 286). Despite the protective role of the astrocytic glial scar in acute SCI, its evolution and persistence in the sub-acute and chronic stages of injury has been considered as a potent inhibitor for spinal cord repair and regeneration (267, 287). A number of inhibitory molecules have been associated with activated astrocytes and their secreted products such as proteoglycans and Tenascin-C (288). Thus, manipulation of the astrocytic scar has been pursued as a promising treatment strategy for SCI (267, 289).

Chondroitin sulfate proteoglycans (CSPGs) are well-known for their contribution to the inhibitory role of the glial scar in axonal regeneration (290–295), sprouting (296–299), conduction (300–302), and remyelination (241, 303–307). In normal condition, basal levels of CSPGs are expressed in the CNS that play critical roles in neuronal guidance and synapse stabilization (90, 308). Following injury, CSPGs (neurocan, versican, brevican, and phosphacan) are robustly upregulated and reach their peak of expression at 2 weeks post-SCI and remain upregulated chronically (309, 310). Mechanistically, disruption of BSB and hemorrhage following traumatic SCI triggers upregulation of CSPGs in the glial scar by exposing the scar forming cells to factors in plasma such as fibrinogen (311). Studies in cortical injury have shown that fibrinogen induces CSPG expression in astrocytes through TGFβ/Smad2 signaling pathway (311). The authors show that intracellular Smad2 translocation is essential for Smad2 signal transduction process and its inhibition reduces scar formation (312). In contrast, another study has identified that TGFβ induces CSPGs production in astrocytes through a SMAD independent pathway (313). This study showed a significant upregulation of CSPGs in SMAD2 and SMAD4 knockdown astrocytes. Interestingly, CSPG upregulation was found to be mediated by the activation of the phosphoinositide 3-kinase (PI3K)/Akt and mTOR axis (313). Further studies are required to confirm these findings.

Extensive research in the past few decades has demonstrated the inhibitory effect of CSPGs on axon regeneration (314, 315). The first successful attempt on improving axon outgrowth and/or sprouting by enzymatic degradation of CSPGs using chondroitinase ABC (ChABC) in a rat SCI model was published in 2002 by Bradbury and colleagues (291). This study showed significant improvement in recovery of locomotor and proprioceptive functions following intrathecal delivery of ChABC in a rat model of dorsal column injury (291). This observation was followed by several other studies demonstrating the promise of CSPGs degradation in improvement of axon regeneration and sprouting of the serotonergic (295, 297, 299, 303), sensory (293, 298, 316), corticospinal (291, 297, 303, 317), and rubrospinal fibers (318) in animal models of CNS injury. Additionally, ChABC treatment is shown to be neuroprotective by preventing CSPG induced axonal dieback and degeneration (303, 319, 320). Studies by our group also showed that degradation of CSPGs using ChABC attenuates axonal dieback in corticospinal fibers in chronic SCI model in the rat (303). ChABC also blocks macrophage-mediated axonal degeneration in neural cultures and after SCI (238).

The inhibitory effects of astrocytic glial scar on axonal regeneration has been recently challenged after SCI (321). Using various transgenic mouse models, a study by Sofroniew's and colleagues has shown that spontaneous axon regrowth failed to happen following the ablation or prevention of astrocytic scar in acute and chronic SCI. They demonstrated that when the intrinsic ability of dorsal root ganglion (DRG) neurons for growth was enhanced by pre-conditioning injury as well as local delivery of a combination of axon growth promoting factors into the SCI lesion, the axons grew to the wall of the glial scar and CSPGs within the lesion. However, when astrocyte scarring was attenuated, the pre-conditioned/growth factor stimulated DRG neurons showed a reduced ability for axon growth (321). From these observations, the authors suggested a positive role for the astrocytic scar in axonal regeneration following SCI (321). Overall, this study points to the importance of reactive and scar forming astrocytes and their pivotal role in the repair process following SCI (322). This is indeed in agreement with previous studies by the same group that showed a beneficial role for activated astrocytes in functional recovery after SCI by limiting the speared of infiltrated inflammatory cells and tissue damage in SCI (285). It is also noteworthy that the glial scar is contributed by various cell populations and not exclusively by astrocytes (269, 271). Therefore, the outcomes of this study need to be interpreted in the context of astrocytes and astrocytic scar. Moreover, the reduced capacity of the injured spinal cord for regeneration is not solely driven by the glial scar as other factors including inflammation and damaged myelin play important inhibitory role in axon regeneration (323, 324). Taken together, further investigation is needed to delineate the mechanisms of the glial scar including the contribution of astrocyte-derived factors on axon regeneration in SCI.

### Role of CSPGs on Endogenous Cell Response and Neuroinflammation

While CSPGs were originally identified as an inhibitor of axon growth and plasticity within the glial scar, emerging evidence has also identified them as an important regulator of endogenous cell response. Emerging evidence has identified CSPGs as an inhibitor of oligodendrocytes (241, 272, 306). Replacement of oligodendrocytes is an important repair process in SCI and other demyelinating conditions such as MS (90). SCI and MS triggers activation of endogenous OPCs and their mobilization to the site of injury (143, 162, 306, 325). *In vitro* and *in vivo* evidence shows that CSPGs limit the recruitment of NPCs and OPCs to the lesion and inhibit oligodendrocyte survival, differentiation and maturation (145, 272, 305, 306, 326). Our group and others have shown that targeting CSPGs by ChABC administration or xyloside, or through inhibition of their signaling receptors enhances the capacity of NPCs and OPCs for proliferation, oligodendrocyte differentiation and remyelination following SCI and MS-like lesions (145, 303, 304, 306).

Mechanistically, the inhibitory effects of CSPGs on axon growth and endogenous cell differentiation is mainly governed by signaling through receptor protein tyrosine phosphatase sigma (RPTPσ) and leukocyte common antigen-related phosphatase receptor (LAR) (327). RPTPσ is the main receptor mediating the inhibition of axon growth by CSPGs (327, 328). Improved neuronal regeneration has been demonstrated in RPTPσ–/– mice model of SCI and peripheral nerve injury (328, 329). Blockade of RPTPσ and LAR by intracellular sigma peptide (ISP) and intracellular LAR peptide (ILP), facilitates axon regeneration following SCI (327, 330). Inhibition of RPTPσ results in significant improvement in locomotion and bladder function associated with serotonergic re-innervation below the level of injury in rat SCI (327). Our group has also shown that CSPGs induce caspase-3 mediated apoptosis in NPCs and OPCs *in vitro* and in oligodendrocytes in the injured spinal cord that is mediated by both RPTPσ and LAR (241). Inhibition of LAR and RPTPσ sufficiently attenuates CSPG-mediated inhibition of oligodendrocyte maturation and myelination *in vitro* and attenuated oligodendrocyte cell death after SCI (241).

CSPGs have been implicated in regulating immune response in CNS injury and disease. Interestingly, our recent studies indicated that CSPGs signaling appears to restrict endogenous repair by promoting a pro-inflammatory immune response in SCI (241, 331). Inhibition of LAR and RPTPσ enhanced an anti-inflammatory environment after SCI by promoting the populations of pro-regenerative M2-like microglia/macrophages and regulatory T cells (241) that are known to promote repair process (224). These findings are also in agreement with recent studies in animal models of MS that unraveled a pro-inflammatory role for CSPGs in autoimmune demyelinating conditions (332). In MS and EAE, studies by Stephenson and colleagues have shown that CSPGs are abundant within “the leucocyte-containing perivascular cuff,” the entry point of inflammatory cells to the CNS tissue (332). Presence of CSPGs in these perivascular cuffs promotes “trafficking” of immune cells to induce a pro-inflammatory response in MS condition. In contrast to these new findings, early studies in SCI described that preventing CSPG formation with xyloside treatment at the time of injury results in poor functional outcome, while manipulation of CSPGs at 2 days after SCI was beneficial for functional recovery (333). These differential outcomes were associated with the modulatory role of CSPGs in regulating the response of macrophages/microglia. Disruption in CSPG formation immediately after injury promoted an M1 pro-inflammatory phenotype in macrophages/microglia, whereas delayed manipulation of CSPGs resulted in a pro-regenerative M2 phenotype (333). In EAE, by products of CSPG degradation also improve the outcomes by attenuating T cell infiltration and their expression of pro-inflammatory cytokines IFN-γ and TNFα (334).

These emerging findings suggest an important immunomodulatory role for CSPGs in CNS injury and disease; further investigations are needed to elucidate CSPG mechanisms in regulating neuroinflammation. Altogether, current evidence has identified a multifaceted inhibitory role for CSPGs in regulating endogenous repair mechanisms after SCI, suggesting that targeting CSPGs may present a promising treatment strategy for SCI.

## Concluding Remarks

Traumatic SCI represents a heterogeneous and complex pathophysiology. While pre-clinical research on SCI has been an ongoing endeavor for over a century, our understanding of SCI mechanisms has been increased remarkably over the past few decades. This is mainly due to the development of new transgenic and preclinical animal models that has facilitated rapid discoveries in SCI mechanisms. Although SCI research has made an impressive advancement, much work is still needed to translate the gained knowledge from animal studies to clinical applications in humans.

## Author Contributions

AA, SD, and SK-A have all contributed to literature review and writing this manuscript. AA and SK-A contributed to the production of figures. All authors have approved the final version of the manuscript.

### Conflict of Interest Statement

The authors declare that the research was conducted in the absence of any commercial or financial relationships that could be construed as a potential conflict of interest.
